# A radiogenomic dataset of non-small cell lung cancer

**DOI:** 10.1038/sdata.2018.202

**Published:** 2018-10-16

**Authors:** Shaimaa Bakr, Olivier Gevaert, Sebastian Echegaray, Kelsey Ayers, Mu Zhou, Majid Shafiq, Hong Zheng, Jalen Anthony Benson, Weiruo Zhang, Ann N. C. Leung, Michael Kadoch, Chuong D. Hoang, Joseph Shrager, Andrew Quon, Daniel L. Rubin, Sylvia K. Plevritis, Sandy Napel

**Affiliations:** 1 Department of Electrical Engineering, Stanford University, Stanford, CA 94305, USA; 2 Department of Medicine, Stanford University School of Medicine, Stanford, CA 94305, USA; 3 Department of Radiology, Stanford University School of Medicine, Stanford, CA 94305, USA; 4 Department of Cardiothoracic Surgery, Stanford University School of Medicine, Stanford, CA 94305, USA; 5 Department of Medicine, Johns Hopkins University, 733 N Broadway, Baltimore, MD 21205, USA; 6 Department of Radiology, University of California Davis, Sacramento, CA 95817, USA; 7 Thoracic and GI Oncology Branch, National Institutes of Health/National Cancer Institute, MD 20892, USA; 8 Stanford School of Medicine, Division of Thoracic Surgery, Department of Cardiothoracic Surgery, Stanford, CA 94305, USA; 9 VA Palo Alto Health Care System, CA 94304, USA

**Keywords:** Prognostic markers, Cancer genomics, Cancer imaging, Computational models

## Abstract

Medical image biomarkers of cancer promise improvements in patient care through advances in precision medicine. Compared to genomic biomarkers, image biomarkers provide the advantages of being non-invasive, and characterizing a heterogeneous tumor in its entirety, as opposed to limited tissue available via biopsy. We developed a unique radiogenomic dataset from a Non-Small Cell Lung Cancer (NSCLC) cohort of 211 subjects. The dataset comprises Computed Tomography (CT), Positron Emission Tomography (PET)/CT images, semantic annotations of the tumors as observed on the medical images using a controlled vocabulary, and segmentation maps of tumors in the CT scans. Imaging data are also paired with results of gene mutation analyses, gene expression microarrays and RNA sequencing data from samples of surgically excised tumor tissue, and clinical data, including survival outcomes. This dataset was created to facilitate the discovery of the underlying relationship between tumor molecular and medical image features, as well as the development and evaluation of prognostic medical image biomarkers.

## Background and Summary

Advances in high-throughput molecular technologies hold great promise for the development of genomic biomarkers that enable precision medicine tailored to specific patients. These molecular biomarkers deliver powerful diagnostic information, as well as high prognostic significance. Similarly, medical imaging technologies provide tools for measuring the structural, functional and physiologic properties of tissue. Identifying image-based properties of tumors through medical images is a standard part of diagnosis, clinical staging, and treatment planning. Because image interpretation can be subjective, for medical imaging to have a role in personalized medicine, the development of robust, standardized image features that can be used to predict molecular properties, prognosis and/or treatment response, is required. These standardized features can be in the form of semantic annotations acquired from human observers, or radiomic features, i.e. quantitative image features computed from the image pixels. Quantitative image features include tumor size and shape, image intensity distributions, and image texture. While the adoption of molecular technologies can be limited by cost and the invasiveness of the procedure, medical imaging is, more commonly, part of the standard of care^1^. Moreover, in comparison to molecular profiling, radiomic characterization provides a more comprehensive representation of the tumor. Since molecular profiling is restricted to the region of the biopsy, it results in an incomplete representation of the heterogeneous tissue of the tumor. On the other hand, molecular technologies allow profiling of genes expressed in the tissue sample. This complementary relationship suggests that combining the use of molecular and imaging biomarkers has the potential to improve patient care and to provide insight into how molecular mechanisms give rise to imaging phenotypes.

The prognostic power of medical image features and their link to molecular properties has only been recently investigated for certain cancer types^2–20^. An important challenge in such radiogenomic studies is the scarcity of large data sets containing medical images, extracted image features, gene expression profiles, and clinical data with survival outcomes. Specifically, for NSCLC, which is the leading cause of cancer death^21^, there is a dearth of available datasets that contain medical images, molecular features, and associated clinical data. In NSCLC, CT and PET/CT are the investigation tools of choice for diagnosis, staging and monitoring of response to treatment. From these scans, one can compute a large number of quantitative image features for associations with tumor molecular features and clinical outcomes. Molecular profiles of tumors can be obtained through needle biopsies or samples of surgically-excised tumors. Clinical data and outcomes can be obtained from standard medical follow-up. While large molecular datasets with clinical data are readily available^22–25^, there are fewer public medical imaging datasets combined with clinical and molecular data. For example, while five independent NSCLC datasets containing collectively 788 subjects were used in a radiogenomics study^7^, only 89 subjects had imaging, molecular and clinical data. Moreover, that dataset included CT scans but did not contain PET/CT data. It is important to continue to create large integrated databases available for discovery and validation of biomarkers, and so we created this dataset to allow researchers to investigate the relationships between image features, tumor molecular phenotype, and survival outcomes.

Between 2008 and 2012, we collected clinical and imaging data for 211 subjects referred for surgical treatment and obtained tissue samples from the excised tumors, where available. Tissue samples were analyzed to produce molecular phenotypes using gene microarrays, RNA sequencing technology, or both, in addition to standard-of-care NSCLC mutational testing. We also collected clinical data, such as: age, gender, weight, ethnicity, smoking status, TNM stage, histopathological grade. In addition, we included 3D tumor segmentations of the CT studies that were used for computation of 3D quantitative image features. Not all data are available for all subjects due to limitations in resources; out of the 211 subjects, 116 have all data types expect for micro-array (the data type with the smallest number of subjects), 130 have clinical, imaging (CT and PET/CT), and molecular (RNA-Seq) as detailed in Tables 1 and 2.

## Methods

### Subject Demographics and Clinical Data

With approval of our respective Institutional Review Boards (IRB), we recruited a total of 211 subjects for the following two cohorts: (1) The R01 cohort consisted of 162 NSCLC subjects (38 females, 124 males, age at scan: mean 68, range 42–86) from Stanford University School of Medicine (69) and Palo Alto Veterans Affairs Healthcare System (93). Subjects were recruited between April 7^th^, 2008 and September 15^th^, 2012. Subjects signed written consent forms according to the guidelines of institutions’ IRBs. The subjects were selected from a pool of early stage NSCLC patients, referred for surgical treatment with preoperative CT and PET/CT performed prior to surgical procedures. Samples of excised tissues were later used to obtain mutation data and gene expression data using gene expression microarrays, or RNA sequencing, or both. Identifiers for this set of 162 subjects are in the format R01-XXXXXX. (2) The AMC cohort, consisting of 49 additional subjects (33 females, 16 males, age at scan: mean 67, range 24–80), was retrospectively collected from Stanford University School of Medicine based on the same criteria in addition to the availability of the following clinical mutational test results: Epidermal Growth Factor Receptor (EGFR), Kirsten Rat Sarcoma viral oncogene homolog (KRAS), and Anaplastic Lymphoma Kinase (ALK). Identifiers for this set of 49 subjects are in the format AMC-XXXXXX. For both cohorts, clinical data included, where available, smoking history (211), survival (211), recurrence status (210), histology (211), histopathological grading (162) and Pathological TNM staging (161). There were 172 adenocarcinomas and 35 squamous cell carcinomas and 4 not otherwise specified with grades ranging from poorly to well-differentiated. Clinical date features (e.g. recurrence date and scan dates) are shifted for anonymization purposes and are chronologically ordered relative to each other. Table 3 summarizes clinical data of the cohorts, and Table 4 lists all clinical features.

### Imaging Data

Subjects received preoperative CT and PET/CT scans at Stanford University Medical Center and Palo Alto Veterans Affairs Healthcare System prior to surgical treatment as part of their care. Different scanners were used depending on the institution and physician choice and scanning protocols also varied.

#### De-Identification of Imaging Data

All imaging data were de-identified prior to analysis at Stanford. For subjects from Stanford, we de-identified the imaging data using the Medical Imaging Resource Center (MIRC) Clinical Trial Processor (CTP) (RSNA, Oakbrook, IL). The MIRC CTP is a software tool designed to Anonymize DICOM objects to remove protected health information. Medical image data from VA subjects were de-identified using PACSGEAR (Perceptive Software, Pleasanton, CA).

Prior to making the data available on The Cancer Imaging Archive (TCIA)^26^, we performed a second round of de-identification using CTP, further assuring complete removal of any identifying information. TCIA complies with HIPAA de-identification standards using the Safe Harbor Method as defined in section 164.514(b)(2) of the HIPPA Privacy Rule. This is done by incorporating the “Basic Application Confidentiality Profile” which is amended by inclusion of the following profile options: Clean Pixel Data Option, Clean Descriptors Option, Retain Longitudinal with Modified Dates Option, Retain Patient Characteristics Option, Retain Device Identity Option, and Retain Safe Private Option. The de-identification rules applied to each object are recorded by TCIA in the DICOM sequence Method Code Sequence [0012,0063] by entering the Code Value, Coding Scheme Designator, and Code Meaning for each profile and option that were applied to the DICOM object during de-identification^27^.

#### CT Data

CT images in DICOM format^28^ are available from 211 subjects. Since this is a retrospectively collected dataset, different subjects were scanned using different scanners, scanning protocols and scanning parameters: slice thickness of 0.625–3 mm (median: 1.5 mm) and an X-ray tube current of 124–699 mA (mean 220 mA) at 80–140 kVp (mean 120 kVp). Detailed scanning parameters, including scanner make and model are specified in the DICOM headers. Scans were acquired with subjects in supine position with arms at sides, from the apex of the lung to the adrenal gland within a single breath-hold. Table 5 summarizes the ranges of CT parameters used for our cohort.

#### PET/CT Data

Fasting Fluorodeoxyglucose ^18^F-FDG PET/CT data are available for 201 subjects. A GE Discovery D690 PET/CT was used for PET/CT scanning at Stanford University Medical Center, while the Palo Alto VA employed a GE Discovery PET/CT scanner. (The exact model of PET/CT scanners are specified DICOM image headers.) FDG Dose and uptake time were 138.90–572.25 MBq (mean 309.26 MBq) and 23.08–128.90 min (mean 66.58 minutes), respectively. PET images were generated at both sites using a similar protocol. Specifically, CT-based attenuation correction was utilized with iterative Ordered Subset Expectation Maximization (OSEM) reconstruction. Image acquisition included routine coverage of base-of-skull to mid-thigh with additional spot views where necessary. Each bed position was 1–5-minute acquisition, dependent on su weight. Table 6 summarizes ranges of scan parameters used to obtain PET/CT images. This PET/CT data set was used to identify tumor PET-FDG uptake features associated with gene expression signatures and survival^29^.

#### CT and PET/CT acquisition protocols

It has been recognized that the results of quantitative analyses (including e.g., radiomics) of images will vary as a function of image acquisition and reconstruction protocol^30–38^. However, we note that the imaging datasets reported here were acquired over several years and from several institutions, and not as part of a prospective trial. For these reasons there was no attempt to harmonize the acquisition and reconstruction protocols.

#### Semantic Annotations

Semantic annotations are available for axial CT series of 190 subjects. The template of semantic terms was developed in consensus by two academic thoracic radiologists (A.N.C.L. and D.A.) with expertise and interest in lung cancer imaging. The template was developed for nodules as they are the most common manifestation of lung cancer. As a result, we provide no semantic annotations for cancers of other manifestations, e.g., central obstructive tumors or "pneumonic tumors”. The template contains 28 nodule analysis features and parenchymal features comprising conventional and newly developed features used for diagnosis and staging using the CT images. Nodule features describe anatomy location, geometry, internal features and other associated findings of the nodules.

Parenchymal features characterize lung emphysema, bronchi and lumen. The selected terms are in common usage in radiology clinical practice and are derived from descriptions in the radiology literature; definitions of some of these, such as “nodule” are found in the Fleischner Society: Glossary of Terms for Thoracic Imaging^39^. Table 7 (available online only) describes the semantic features included in the template. The ePAD template that we developed forces complete annotation for each nodule, resulting in all applicable features being collected. There are some features whose presence are conditioned upon other features being present. For example, the primary emphysema pattern feature is not collected when emphysema is not present in the lung. ePAD creates annotations in the Annotation and Image Mark-up (AIM) file format using a controlled vocabulary. The AIM information model is designed to be semantically inoperable. Information such as annotator identity, annotation date, and a reference to the annotated image, complement information on anatomic entities and imaging observation characteristics of the referenced image. AIM files supplement DICOM and other image formats which do not contain information on the meaning of the pixels in the image^40,41^. One radiologist (A.N.L.) with more than 20 years of experience ascribed the semantic annotations for all subjects’ CT scans using ePAD, an open-source and freely available web-based quantitative imaging informatics platform^41^. While we acknowledge that semantic annotations are subjective and subject to intra-and inter-reader variability, these were used in several studies, e.g., to predict EGFR and KRAS mutation status^42^, and to create a radiogenomic map linking semantic features to gene expression profiles generated by RNA sequencing^13^.

#### Segmentations

Initial segmentations for 144 subjects were obtained from an axial CT image series using an unpublished automatic segmentation algorithm. All of these segmentations were viewed by a thoracic radiologist (M.K.) with more than 5 years of experience and edited as necessary using ePAD. Final segmentations were reviewed by an additional thoracic radiologist (A.N.L.); disagreements in tumor boundaries were discussed and edited as appropriate, with final approval by A.N.L. All segmentations are stored as DICOM Segmentation Objects^28^.

### Molecular Data

#### Tumor Preparation

All tumor samples were collected from treatment-naïve subjects during surgical procedure. Following excision, the surgeon cut a 3–5-mm-thick slice along the longest axis of the excised tissue, which was frozen within 30 minutes of excision. It was later retrieved for RNA extraction. Molecular data are available from EGFR, KRAS, ALK mutational testing, gene expression microarrays, and RNA sequencing. Tumors from 17 subjects were analyzed using both gene expression microarrays and RNA sequencing.

#### Mutational testing

EGFR, KRAS and ALK mutation status are available from clinical records in 206, 205, and 196 subjects, respectively. Single nucleotide mutation detection was performed using SNaPshot technology based on dideoxy single-base extension of oligonucleotide primers after multiplex polymerase chain reaction (PCR). Exons 18, 19, 20 and 21 were tested for EGFR mutations. Exon 2 Positions 12 and 13 were tested for missense KRAS mutations with amino acid substitution. Mutation results were a combination of mutation at any location of the tested exons. For ALK, EML4-ALK translocation detection test was performed using fluorescence in situ hybridization (FISH).

#### Gene Expression Microarray Data

Gene expression microarray data was collected for the subset of 26 subjects, who underwent surgical treatment between April 7, 2008 and May 21, 2010. RNA was processed at the Stanford Functional Genomics Facility using Illumina Whole Genome Bead Chips (Human HT-12; Illumina, San Diego, CA). These data were preprocessed as follows: First, we filtered the microarray probes on the basis of a significant detection call in at least 60% of the samples. Next, we log transformed the microarray data and used quantile normalization to normalize between arrays. These data, along with the corresponding CT images, were used to describe associations between image features, gene expression, and survival^10,29^.

#### RNA Sequencing Data

Based on availability and quality of available tissue, RNA sequencing was performed on samples from 130 subjects (17 of which intersect with the gene expression microarray dataset described in the previous section). We excluded RNASeq for tissue samples with RNA integrity number (RIN) below 2.5. Total RNA was extracted from the tissue samples and converted into a library for paired-end sequencing on Illumina Hiseq according to the protocol for the Illumina TruSeq Sample preparation kit (Centrillion Biosciences, Palo Alto, CA). Briefly, total RNA quality and quantity were measured by BioAnalyzer (Agilent). For library preparation, the TruSeq Total Stranded RNA with Ribo-Zero Reduction (Illumina) was used following manufacturer’s instructions. This method includes a Ribo-Zero rRNA depletion step, followed by fragmentation and cDNA synthesis using SuperScript II (Life Technologies). The cDNA was A-tailed, ligated and amplified using the materials in the TruSeq Total Stranded RNA with Ribo-Zero Reduction kit. Quality was confirmed using the BioAnalyzer and finally the concentration evaluated by KAPA qPCR (KAPA Biosystems). Prior to sequencing, samples were diluted to 4 nmol and pooled. Pooled libraries were clustered via the cBOT and sequenced on the HiSeq 2500 (illumina) following manufacturer’s instructions. The set of 130 tissue samples was sequenced in three batches of sizes 16, 66, 48.

Data processing was performed by Centrillion Biosciences as follows: reads were aligned to the human genome (hg19) using the alignment algorithm STAR^43^ version 2.3 with 91 bases of splice junction overhangs. Next, Cufflinks version 2.0.2^44^ was used to determine the expression calls in each sample using Fragments Per Kilobase of transcript per Million mapped reads (FPKM).

## Data Records

### Subject Identifiers

A unique identifier for each subject is identical in all four public data records in this dataset. Subject ID’s are 6-digit numbers in the form of R01-XXXXXX or AMC-XXXXXX.

#### Data Record 1

Clinical, image, semantic data for all subjects are stored in The Cancer Imaging Archive (TCIA) (Data Citation 1). One comma-delimited file contains clinical data for all subjects with unique subject identifiers. Semantic features for each subject are stored in Annotation and Image Markup (AIM) files^45^. CT and PET/CT Images are in DICOM format. Where available, segmentations are provided as DICOM Segmentation Objects.

#### Data Record 2

Image data of 26 subjects had been previously deposited in the TCIA repository (Data Citation 2). These images were given new subject names in the form R01-XXXXXX as part of the new dataset described in this work.

#### Data Record 3

Gene expression microarray data, available for 26 subjects, were deposited in National Center for Biotechnology Information Gene Expression Omnibus (NCBI GEO)^46^ (Data Citation 3). The subject identifiers are identical to subject names in Data Record 2. Processed gene clusters were deposited in tab-delimited files with column values corresponding to microarray ID, log2 transformed quantile normalized and probe selection detection-p-value, respectively. This data record also contains raw expression data, as well as matrix data obtained prior to normalization.

#### Data Record 4

Raw and processed sequencing data obtained from RNASeq for 130 subjects are available at NCBI GEO (Data Citation 4). The subject IDs are identical to subject names in Data Record 1.

## Technical Validation

All CT and PET/CT data were collected as part of patient care and therefore all quality assurance was performed by the institution that collected the data.

## Usage Notes

All data are freely available to browse, download, and use for commercial, scientific and educational purposes as outlined in the Creative Commons Attribution 3.0 Unported License. Users should properly cite this source for any work based on this dataset.

## Additional information

**How to cite this article:** Bakr, S. *et al.* A radiogenomic dataset of non-small cell lung cancer. Sci. Data. 5:180202 doi: 10.1038/sdata.2018.202 (2018).

**Publisher’s note:** Springer Nature remains neutral with regard to jurisdictional claims in published maps and institutional affiliations.

## Supplementary Material



## Figures and Tables

**Table 1:** Summary of the major collected data types and the corresponding number of subjects with available data.

**Data Type**	**Number of subjects**
Clinical Data	211
CT	211
CT Tumor Segmentations	144
CT Semantic Annotations	190
PET/CT	201
RNA-Seq	130
Gene expression Microarrays	26

**Table 2:** Subject IDs versus data type. Yes/no indicates if a data type is available for that particular subject.

	**Clinical**	**CT**	**PET/CT**	**RNA-Seq**	**Semantic Annotations**	**Segmentations**
AMC 001	Yes	Yes	Yes	No	No	No
AMC 002	Yes	Yes	No	No	No	No
AMC 003	Yes	Yes	Yes	No	Yes	No
AMC 004	Yes	Yes	Yes	No	No	No
AMC 005	Yes	Yes	No	No	Yes	No
AMC 006	Yes	Yes	Yes	No	Yes	No
AMC 007	Yes	Yes	No	No	Yes	No
AMC 008	Yes	Yes	No	No	Yes	No
AMC 009	Yes	Yes	Yes	No	Yes	No
AMC 010	Yes	Yes	Yes	No	Yes	No
AMC 011	Yes	Yes	Yes	No	No	No
AMC 012	Yes	Yes	Yes	No	No	No
AMC 013	Yes	Yes	Yes	No	Yes	No
AMC 014	Yes	Yes	Yes	No	Yes	No
AMC 015	Yes	Yes	Yes	No	Yes	No
AMC 016	Yes	Yes	Yes	No	Yes	No
AMC 017	Yes	Yes	Yes	No	No	No
AMC 018	Yes	Yes	Yes	No	Yes	No
AMC 019	Yes	Yes	No	No	Yes	No
AMC 020	Yes	Yes	Yes	No	Yes	No
AMC 021	Yes	Yes	Yes	No	Yes	No
AMC 022	Yes	Yes	Yes	No	Yes	No
AMC 023	Yes	Yes	Yes	No	Yes	No
AMC 024	Yes	Yes	Yes	No	Yes	No
AMC 025	Yes	Yes	Yes	No	No	No
AMC 026	Yes	Yes	Yes	No	Yes	No
AMC 027	Yes	Yes	Yes	No	Yes	No
AMC 028	Yes	Yes	No	No	Yes	No
AMC 029	Yes	Yes	No	No	Yes	No
AMC 030	Yes	Yes	Yes	No	Yes	No
AMC 031	Yes	Yes	No	No	Yes	No
AMC 032	Yes	Yes	Yes	No	Yes	No
AMC 033	Yes	Yes	Yes	No	Yes	No
AMC 034	Yes	Yes	No	No	Yes	No
AMC 035	Yes	Yes	Yes	No	Yes	No
AMC 036	Yes	Yes	Yes	No	Yes	No
AMC 037	Yes	Yes	Yes	No	Yes	No
AMC 038	Yes	Yes	Yes	No	Yes	No
AMC 039	Yes	Yes	Yes	No	Yes	No
AMC 040	Yes	Yes	Yes	No	Yes	No
AMC 041	Yes	Yes	Yes	No	Yes	No
AMC 042	Yes	Yes	Yes	No	Yes	No
AMC 043	Yes	Yes	No	No	Yes	No
AMC 044	Yes	Yes	Yes	No	Yes	No
AMC 045	Yes	Yes	Yes	No	Yes	No
AMC 046	Yes	Yes	Yes	No	Yes	No
AMC 047	Yes	Yes	Yes	No	Yes	No
AMC 048	Yes	Yes	Yes	No	Yes	No
AMC 049	Yes	Yes	Yes	No	Yes	No
R01 001	Yes	Yes	Yes	No	Yes	Yes
R01 002	Yes	Yes	Yes	No	Yes	Yes
R01 003	Yes	Yes	Yes	Yes	Yes	Yes
R01 004	Yes	Yes	Yes	Yes	Yes	Yes
R01 005	Yes	Yes	Yes	Yes	Yes	Yes
R01 006	Yes	Yes	Yes	Yes	Yes	Yes
R01 007	Yes	Yes	Yes	Yes	Yes	Yes
R01 008	Yes	Yes	Yes	No	Yes	Yes
R01 009	Yes	Yes	Yes	No	No	No
R01 010	Yes	Yes	Yes	No	Yes	Yes
R01 011	Yes	Yes	Yes	No	Yes	Yes
R01 012	Yes	Yes	Yes	Yes	Yes	Yes
R01 013	Yes	Yes	Yes	Yes	Yes	Yes
R01 014	Yes	Yes	Yes	Yes	Yes	Yes
R01 015	Yes	Yes	Yes	Yes	Yes	Yes
R01 016	Yes	Yes	Yes	Yes	Yes	Yes
R01 017	Yes	Yes	Yes	Yes	Yes	Yes
R01 018	Yes	Yes	Yes	Yes	Yes	Yes
R01 019	Yes	Yes	Yes	No	Yes	Yes
R01 020	Yes	Yes	Yes	No	Yes	Yes
R01 021	Yes	Yes	Yes	Yes	Yes	Yes
R01 022	Yes	Yes	Yes	Yes	Yes	Yes
R01 023	Yes	Yes	Yes	Yes	Yes	Yes
R01 024	Yes	Yes	Yes	Yes	Yes	Yes
R01 025	Yes	Yes	Yes	No	Yes	Yes
R01 026	Yes	Yes	Yes	Yes	Yes	Yes
R01 027	Yes	Yes	Yes	Yes	Yes	Yes
R01 028	Yes	Yes	Yes	Yes	Yes	Yes
R01 029	Yes	Yes	Yes	Yes	Yes	Yes
R01 030	Yes	Yes	Yes	No	Yes	Yes
R01 031	Yes	Yes	Yes	Yes	Yes	Yes
R01 032	Yes	Yes	Yes	Yes	Yes	Yes
R01 033	Yes	Yes	Yes	Yes	Yes	Yes
R01 034	Yes	Yes	Yes	Yes	Yes	Yes
R01 035	Yes	Yes	Yes	Yes	Yes	Yes
R01 036	Yes	Yes	Yes	No	Yes	Yes
R01 037	Yes	Yes	Yes	Yes	Yes	Yes
R01 038	Yes	Yes	Yes	Yes	Yes	Yes
R01 039	Yes	Yes	Yes	Yes	Yes	Yes
R01 040	Yes	Yes	Yes	Yes	Yes	Yes
R01 041	Yes	Yes	Yes	Yes	Yes	Yes
R01 042	Yes	Yes	Yes	Yes	Yes	Yes
R01 043	Yes	Yes	Yes	Yes	Yes	Yes
R01 044	Yes	Yes	Yes	No	Yes	Yes
R01 045	Yes	Yes	Yes	No	Yes	Yes
R01 046	Yes	Yes	Yes	Yes	Yes	Yes
R01 047	Yes	Yes	Yes	No	Yes	Yes
R01 048	Yes	Yes	Yes	Yes	Yes	Yes
R01 049	Yes	Yes	Yes	Yes	Yes	Yes
R01 050	Yes	Yes	Yes	No	Yes	Yes
R01 051	Yes	Yes	Yes	Yes	Yes	Yes
R01 052	Yes	Yes	Yes	Yes	Yes	Yes
R01 053	Yes	Yes	Yes	No	Yes	Yes
R01 054	Yes	Yes	Yes	Yes	Yes	Yes
R01 055	Yes	Yes	Yes	Yes	Yes	Yes
R01 056	Yes	Yes	Yes	Yes	Yes	Yes
R01 057	Yes	Yes	Yes	Yes	Yes	Yes
R01 058	Yes	Yes	Yes	No	Yes	Yes
R01 059	Yes	Yes	Yes	Yes	Yes	Yes
R01 060	Yes	Yes	Yes	Yes	Yes	Yes
R01 061	Yes	Yes	Yes	Yes	Yes	Yes
R01 062	Yes	Yes	Yes	Yes	Yes	Yes
R01 063	Yes	Yes	Yes	Yes	Yes	Yes
R01 064	Yes	Yes	Yes	Yes	Yes	Yes
R01 065	Yes	Yes	Yes	Yes	Yes	Yes
R01 066	Yes	Yes	Yes	Yes	Yes	Yes
R01 067	Yes	Yes	Yes	Yes	Yes	Yes
R01 068	Yes	Yes	Yes	Yes	Yes	Yes
R01 069	Yes	Yes	Yes	Yes	Yes	Yes
R01 070	Yes	Yes	Yes	No	Yes	Yes
R01 071	Yes	Yes	Yes	Yes	Yes	Yes
R01 072	Yes	Yes	Yes	Yes	Yes	Yes
R01 073	Yes	Yes	Yes	Yes	Yes	Yes
R01 074	Yes	Yes	Yes	No	Yes	Yes
R01 075	Yes	Yes	Yes	No	Yes	Yes
R01 076	Yes	Yes	Yes	Yes	Yes	Yes
R01 077	Yes	Yes	Yes	Yes	Yes	Yes
R01 078	Yes	Yes	Yes	Yes	Yes	Yes
R01 079	Yes	Yes	Yes	Yes	Yes	Yes
R01 080	Yes	Yes	Yes	Yes	Yes	Yes
R01 081	Yes	Yes	Yes	Yes	Yes	Yes
R01 082	Yes	Yes	Yes	No	Yes	Yes
R01 083	Yes	Yes	Yes	Yes	Yes	Yes
R01 084	Yes	Yes	Yes	Yes	Yes	Yes
R01 085	Yes	Yes	Yes	No	Yes	Yes
R01 086	Yes	Yes	Yes	No	Yes	Yes
R01 087	Yes	Yes	Yes	No	Yes	Yes
R01 088	Yes	Yes	Yes	No	Yes	Yes
R01 089	Yes	Yes	Yes	Yes	Yes	Yes
R01 090	Yes	Yes	Yes	No	Yes	Yes
R01 091	Yes	Yes	Yes	Yes	Yes	Yes
R01 092	Yes	Yes	Yes	No	Yes	Yes
R01 093	Yes	Yes	Yes	Yes	Yes	Yes
R01 094	Yes	Yes	Yes	Yes	Yes	Yes
R01 095	Yes	Yes	Yes	No	Yes	Yes
R01 096	Yes	Yes	Yes	Yes	Yes	Yes
R01 097	Yes	Yes	Yes	Yes	Yes	Yes
R01 098	Yes	Yes	Yes	Yes	Yes	Yes
R01 099	Yes	Yes	Yes	Yes	Yes	Yes
R01 100	Yes	Yes	Yes	Yes	Yes	Yes
R01 101	Yes	Yes	Yes	Yes	Yes	Yes
R01 102	Yes	Yes	Yes	Yes	Yes	Yes
R01 103	Yes	Yes	Yes	Yes	Yes	Yes
R01 104	Yes	Yes	Yes	Yes	Yes	Yes
R01 105	Yes	Yes	Yes	Yes	Yes	Yes
R01 106	Yes	Yes	Yes	Yes	Yes	Yes
R01 107	Yes	Yes	Yes	Yes	Yes	Yes
R01 108	Yes	Yes	Yes	Yes	Yes	Yes
R01 109	Yes	Yes	Yes	Yes	Yes	Yes
R01 110	Yes	Yes	Yes	Yes	Yes	Yes
R01 111	Yes	Yes	Yes	Yes	Yes	Yes
R01 112	Yes	Yes	Yes	Yes	Yes	Yes
R01 113	Yes	Yes	Yes	Yes	Yes	Yes
R01 114	Yes	Yes	Yes	Yes	Yes	Yes
R01 115	Yes	Yes	Yes	Yes	Yes	Yes
R01 116	Yes	Yes	Yes	Yes	Yes	Yes
R01 117	Yes	Yes	Yes	Yes	Yes	Yes
R01 118	Yes	Yes	Yes	Yes	Yes	Yes
R01 119	Yes	Yes	Yes	Yes	Yes	Yes
R01 120	Yes	Yes	Yes	Yes	Yes	Yes
R01 121	Yes	Yes	Yes	Yes	Yes	Yes
R01 122	Yes	Yes	Yes	Yes	Yes	Yes
R01 123	Yes	Yes	Yes	Yes	Yes	Yes
R01 124	Yes	Yes	Yes	Yes	Yes	Yes
R01 125	Yes	Yes	Yes	Yes	Yes	Yes
R01 126	Yes	Yes	Yes	Yes	Yes	Yes
R01 127	Yes	Yes	Yes	Yes	Yes	Yes
R01 128	Yes	Yes	Yes	Yes	Yes	Yes
R01 129	Yes	Yes	Yes	Yes	Yes	Yes
R01 130	Yes	Yes	Yes	Yes	Yes	Yes
R01 131	Yes	Yes	Yes	Yes	Yes	Yes
R01 132	Yes	Yes	Yes	Yes	Yes	Yes
R01 133	Yes	Yes	Yes	Yes	No	Yes
R01 134	Yes	Yes	Yes	Yes	Yes	Yes
R01 135	Yes	Yes	Yes	Yes	Yes	Yes
R01 136	Yes	Yes	Yes	Yes	Yes	Yes
R01 137	Yes	Yes	Yes	Yes	Yes	Yes
R01 138	Yes	Yes	Yes	Yes	Yes	Yes
R01 139	Yes	Yes	Yes	Yes	Yes	Yes
R01 140	Yes	Yes	Yes	Yes	Yes	Yes
R01 141	Yes	Yes	Yes	Yes	Yes	Yes
R01 142	Yes	Yes	Yes	Yes	Yes	Yes
R01 143	Yes	Yes	Yes	No	Yes	No
R01 144	Yes	Yes	Yes	Yes	Yes	Yes
R01 145	Yes	Yes	Yes	Yes	Yes	Yes
R01 146	Yes	Yes	Yes	Yes	Yes	Yes
R01 147	Yes	Yes	Yes	No	Yes	No
R01 148	Yes	Yes	Yes	No	No	No
R01 149	Yes	Yes	Yes	No	No	No
R01 150	Yes	Yes	Yes	No	No	No
R01 151	Yes	Yes	Yes	No	Yes	No
R01 152	Yes	Yes	Yes	No	Yes	No
R01 153	Yes	Yes	Yes	No	No	No
R01 154	Yes	Yes	Yes	No	Yes	No
R01 156	Yes	Yes	Yes	No	No	No
R01 157	Yes	Yes	Yes	No	No	No
R01 158	Yes	Yes	Yes	No	No	No
R01 159	Yes	Yes	Yes	No	No	No
R01 160	Yes	Yes	Yes	No	No	No
R01 161	Yes	Yes	Yes	No	No	No
R01 162	Yes	Yes	Yes	No	No	No
R01 163	Yes	Yes	Yes	No	No	No

**Table 3:** Summary of demographic (sex and ethnicity) and clinical cohort characteristics (histology, pathological TNM stage and histopathological grade).

**Feature**	**Number of Subjects**
**Sex**	
Female	76
Male	135
**Ethnicity**	
African-American	6
Asian	24
Caucasian	123
Hispanic/Latino	6
Native Hawaiian/Pacific Islander	3
Not Recorded	49
**Histology**	
Adenocarcinoma	172
Squamous cell carcinoma	35
Not otherwise specified	4
**Pathological T stage**	
T0	0
Tis	6
T1a	40
T1b	31
T1nos	0
T2a	47
T2b	10
T2nos	0
T3	21
T4	7
TX	0
Not Collected	49
**Pathological N stage**	
N0	129
N1	15
N2	18
N3	0
NX	0
Not Collected	49
**Pathological M stage**	
M0	157
M1a	1
M1b	4
Not Collected	49
**Histopathological Grade**	
G1 Well differentiated	32
G2 Moderately differentiated	76
G3 Poorly differentiated	33
Other, Type I: Well to moderately differentiated	9
Other, Type II: Moderately to poorly differentiated	12
Not Collected	49

**Table 4:** List of clinical features collected from subject medical records for our cohort of 211 subjects and corresponding number of patients with filled information for each feature.

**Clinical Features**	**Number of Patients**
Subject affiliation	211
Age at Histological Diagnosis	211
Weight (lbs)	152
Gender	211
Ethnicity	162
Smoking status	211
Pack Years	203
Quit Smoking Year	194
Ground Glass	146
Tumor Location	211
Histology	211
Pathological T stage	162
Pathological N stage	162
Pathological M stage	162
Histopathological Grade	162
Lymphovascular invasion	154
Pleural invasion (elastic, visceral, parietal)	154
EGFR mutation status	206
KRAS mutation status	205
ALK translocation status	196
Adjuvant Treatment	210
Chemotherapy	210
Radiation	210
Recurrence	210
Recurrence Location	210
Date of Recurrence	210
Date of Last Known Alive	211
Survival Status	211
Date of Death	211
CT Date	211
Days between CT and surgery	211
PET Date	162

**Table 5:** Summary of key CT scanning parameters in our cohort.

**Parameter**	**Value**	**No. of Subjects**
Peak kilovoltage (kVp)	100–120	See DICOM image headers for individual scans
X-ray Tube Current (mA)	28–749	See DICOM image headers for individual scans
	0.625	12
	1	64
Slice Thickness (mm)	1.5	114
	2	2
	2.5	15
	3	4

**Table 6:** Summary of key PET/CT parameters in our cohort.

**Parameter**	**Value**
FDG Dose (MBq)	138.90–572.25
FDG uptake time (min)	23.08–128.90

**Table 7:** List of semantic features collected from axial CT for a subset of 190 subjects. Nodule analysis provides information on anatomy location, geometry, internal features and other associated findings of the nodules.

**Nodule Analysis**		
**Category**	**Feature**	**Value**
	Anatomic Location	-Right Upper Lobe-Right Middle Lobe-Right Lower Lobe-Left Upper Lobe-Lingula-Left Lower Lobe-Right bronchial tree-Left bronchial tree-Right side-Left side-Unable to determine
	Axial Location	-Central | Peripheral (edge < 2 cm from visceral pleura)
	Longest diameter (mm)	Integer
	Longest perpendicular diameter (mm)	Integer
	Nodule attenuation	-Solid-Pure Ground Glass (GG) (Non-solid)-Semi-consolidation (attenuation between solid and ground –glass in non-solid nodules)-Part-solid, solid ≤ 5 mm diameter-Part-solid, solid > 5 mm diameter
	Nodule Reticulation	-Absent | -Present (lines inside GG nodule)
Internal Features	Internal Air alveolograms/bronchograms	-Absent | -Present
	Necrosis	-Absent | -Present
	Cavitation	-Absent | -Present
	Nodule Margins – primary pattern	-Smooth (sharply delineated margins – can outline confidently without oscillations or serrations)-Irregular (minor oscillations or serrations of margin)-Lobulated (focal convexity or protrusions of lesion into lung)-Spiculation (several linear radiations of finite length extending into adjacent lung)-Poorly defined (lack of clear delineation of margins – cannot outline confidently)
	Nodule Shape	-Round (roughly spherical)-Oval (ratio of x/y diameters > 1.5)-Complex (neither 1 nor 2)-Polygonal (straight or concave borders)
	Nodule Calcification	-No calcification-Central calcification-Peripheral
Associated Findings	Attachment to Pleura	-Absent | -Present
	Attachment to Vessel	-Absent | -Present
	Attachment to Bronchus	-Absent | -Present
	Pleural Retraction	-Absent | -Present
	Entering Airway	-Absent | -Present
	Thickened adjacent bronchovascular bundle	-Absent | -Present
	Vascular convergence	-Absent | -Present
	Septal thickening	-Absent | -Present
	Nodule Periphery	-Emphysema-Fibrosis (diffuse)-Normal-Scarring (focal)
	Satellite nodules in Primary Lesion Lobe ( ≥ 4 mm, noncalcified)	-Absent | -Solid | -Non-solid | -Semi-consolidation | -Part-solid
	Nodules in NON-lesion lobe SAME Lung ( ≥ 4 mm, noncalcified)	-Absent | -Solid | -Non-solid | -Semi-consolidation | -Part-solid
	Nodules in CONTRALATERAL Lung ( ≥ 4 mm, noncalcified)	-Absent | -Solid | -Non-solid | -Semi-consolidation | -Part-solid
	Centrilobular nodules – diffuse (RB type nodules)	-Absent | -Present
**Lung Parenchyma Analysis**		
**Category**	**Feature**	**Value**
	Emphysema	-Absent | -Present
	Primary emphysema Pattern	-Centrilobular-Pan-acinar-Paraseptal-Paracicatricial-NA
	Primary Distribution	-Upper predominant-Middle Predominant-Lower Predominant-Diffuse, no predominance-Patchy, no predominance-NA or Unable to determine
	Primary Emphysema Laterality	-Right | -Left | -Both
	Secondary Emphysema Pattern	-Centrilobular-Pan-acinar-Paraseptal-Paracicatricial-NA
	Secondary Emphysema Distribution	-Upper predominant-Middle Predominant-Lower Predominant-Diffuse, no predominance-Patchy, no predominance-NA or Unable to determine
	Secondary emphysema laterality	-Right | -Left | -Both
	Overall Emphysema Severity	-None-Low (1-25%)-Moderate (26-50%)-Moderately High (51-75%)-High ( > 75%)
Lung Features	Airway Abnormalities	-Absent | -Present
	Bronchial wall thickening	-Absent | -Present
	Airway ectasia (mild luminal enlargement)	-Absent | -Present
	Bronchiectasis (moderate enlargement)	-Absent | -Present
	Luminal narrowing	-Absent | -Present
	Bronchiolar prominence	-Absent | -Present
	Tree-in-Bud (airway secretions)	-Absent | -Present
	Mosaic oligemia	-Absent | -Present
	Fibrosis	-Absent | -Present
	Anatomic Fibrosis Distribution	-Apical-Upper predominant-Middle Predominant-Lower Predominant-Diffuse, no predominancne-Patchy, no predominance-Unable to determine
	Axial Fibrosis Distribution	-Subpleural-Bronchovascular-Both 1 & 2-Random
	Fibrosis Type	-Usual Interstitial Pneumonia (UIP)-Nonspecific Interstitial Pneumonia (NSIP)-Hypersensitivity Pneumonitis (HP)-Sarcoidosis-Smoking-related-Post-infectious (include Oesophago-Gastro-Duodenoscopy OGD)-Other (specify)-Indeterminate
The “internal” features refer to findings inside (interior of) the tumor. Associated findings are observed within the imaging study but outside the tumor volume. Parenchymal analysis characterizes lung emphysema, bronchi and lumen. The ePAD template used to collect these features requires all features to be collected. However, some features are conditional upon another feature being present. For example, the “primary emphysema pattern” feature is collected unless the “emphysema feature” is present.		
